# Nintedanib alleviates pulmonary inflammation and fibrosis in mice with silicosis

**DOI:** 10.1186/s12890-026-04546-y

**Published:** 2026-07-27

**Authors:** Limin Huang, Li Liu, Liang Tang, Hongxia Tian, Ling Mao

**Affiliations:** 1 Department of Pneumoconiosis, Shanghai Pulmonary Hospital, Tongji University School of Medicine, 507 Zhengmin Road, Yangpu District, Shanghai, 200433 China; 2 2nd People’s Hospital of Shanxi Province, 9 West Kouzhuang Rd., Taiyuan, Shanxi Province 030012 China; 3 Department of Central Laboratory, Shanghai Pulmonary Hospital, School of Medicine, Tongji University, 507 Zhengmin Rd., Shanghai, 200433 China; 4 Department of Respiratory and Pneumoconiosis, 2nd People’s Hospital of Shanxi Province, 9 West Kouzhuang Rd., Taiyuan, Shanxi Province 030012 China

**Keywords:** Nintedanib, Silicosis, Inflammation, Fibrosis, Pulmonary function, Mice

## Abstract

**Objective:**

This study aimed to evaluate nintedanib’s therapeutic effects on mice silicosis by examining pulmonary function, lung inflammation, and fibrosis.

**Methods:**

Mice were categorized into preventive and therapeutic groups, each with control, low-dose, and high-dose treatment subgroups. Cell counts in bronchoalveolar lavage fluid and cytokines in the lung tissue were detected using quantitative polymerase chain reaction. The right ventricular hypertrophy index (RVHI) was assessed, and lung collagen deposition and inflammation were analyzed using various histological and molecular techniques. Forced lung function tests were conducted to evaluate lung compliance, total lung capacity (TLC), and the mouse airway resistance.

**Results:**

In the preventive group, neutrophil counts and levels of pro-inflammatory cytokines in lung tissue were significantly lower in the treatment group than in the control group, with no significant differences in the RVHI. Collagen deposition and pathological scores for inflammation and fibrosis were reduced in the treatment group. In lung function tests, TLC was higher and resistance lower in the high-dose group. In the therapeutic group, cytokine levels and the RVHI were significantly lower in the treatment group, while collagen type I mRNA expression was statistically significant. Lung function tests revealed improved TLC and resistance in the treatment group.

**Conclusion:**

Nintedanib reduces lung inflammation, fibrosis, and right ventricular hypertrophy in mice with silicosis, demonstrating potential therapeutic effects.

**Supplementary Information:**

The online version contains supplementary material available at https://doi.org/10.1186/s12890-026-04546-y.

## Introduction

Silicosis, a diffuse interstitial fibrotic disease of the lungs, is caused by free silica (SiO_2_) dust inhalation. It can progress relentlessly, leading to progressive respiratory insufficiency and failure. Silicosis is one of the most dangerous occupational diseases globally, responsible for approximately 10,000 deaths annually [1], and recent comprehensive analyses, including the 2023 Lancet review and Global Burden of Disease data, confirm a persistent and concerning resurgence driven by modern industrial practices [2, 3]. Despite active prevention efforts, its incidence remains high, particularly with the rise in industries such as artificial stone slabs, denim production, and jewelry polishing.

SiO_2_ particles enter the lungs through the respiratory tract, activating various transcription factors in alveolar macrophages and epithelial cells, a process deeply governed by complex macrophage polarization and phenotypic transition mechanisms [4]. This triggers the release of inflammatory cytokines, leading to the aggregation of neutrophils, lymphocytes, and other immune cells in the lungs, thereby initiating a more extensive inflammatory response [5–7] and causing lung tissue damage [8]. Current treatment primarily focuses on suppressing inflammation and alleviating fibrosis, as no effective curative measures exist, making the active management of silicosis essential.

Nintedanib, a tyrosine kinase inhibitor, targets multiple growth factor receptors, blocking intracellular signaling and regulating cell proliferation [9]. Previous studies have demonstrated that nintedanib induces an anti-inflammatory response and inhibits fibrosis in mouse models of bleomycin-induced pulmonary fibrosis and silicosis [10].

In 2022, the ATS/ERS/JRS/ALAT published guidelines recommending oral nintedanib for antifibrotic treatment in patients with progressive pulmonary fibrosis (PPF) to reduce the risk of lung function decline and exacerbations. Studies have shown that nintedanib slows the rate of lung function decline in patients with moderate-to-severe lung impairment, with a history of dust exposure considered a potential contributory factor to PPF [11], a condition now widely recognized by recent expert consensus as a dynamic and irreversible evolution of interstitial remodeling requiring early intervention [4]. However, of the 663 patients studied, only 39 were exposed to occupational hazards, leaving the specific risk factors unclear [12]. Moreover, a few animal studies have explored the use of nintedanib for treating silicosis in mice, and most have neglected the impact on pulmonary function and pathological changes in lung tissue. Therefore, we evaluated the effects of nintedanib on lung function, inflammation, and fibrosis in silicotic mice from multiple perspectives.

## Materials and methods

### Research subjects

#### Reagents

Crystalline silica particles with a diameter of < 1.3 μm were obtained from Fuhua Mining Technology Co., Ltd. (CAS7631-86-9; purity 99%). These particles were baked at 180 °C for at least 2 h and suspended in sterile, endotoxin-free phosphate-buffered saline (PBS). All silica suspensions were sonicated for a minimum of 15 min and vortexed for 1 min before use. Nintedanib powder, with a purity > 98%, was provided by Boehringer Ingelheim, Germany (CASNO:656247-18-6), batch number 1,103,733, and dissolved in 1% sodium carboxymethyl cellulose (CMC-Na) for administration to mice. The nintedanib suspension was sonicated for 20 min and vortexed for 30 s before use.

#### Animals

Eight-week-old male C57BL/6 mice were kept under specific pathogen-free conditions, with five animals per individually ventilated cage. A total of 70 mice were raised, with 50 used for the study and 20 kept as backups. The mice had free access to irradiated, sterilized dry food and sterile drinking water, with controlled room temperature (18–26 °C) and humidity (40%–70%). Mice were randomly assigned to experimental groups using a computer-generated random number sequence. There were no specific exclusion criteria prior to the experiment. All animals survived the duration of the study, yielding a 100% survival rate, and no animals were excluded from the final analysis. Surgery was performed under isoflurane anesthesia with postoperative analgesia. This study was approved by the Animal Care and Use Committee of Shanghai Pulmonary Hospital, Tongji University (No. K24-181Y) and performed in accordance with ethical guidelines.

#### Silicosis model and nintedanib treatment

The silicosis model was established via a single intratracheal exposure to a silica suspension. Mice were anesthetized with isoflurane, and 20 µL of silica suspension (600 mg of silica in 1 mL of sterile PBS) was injected into the trachea of each mouse.

To establish the preventive model with nintedanib, mice were randomly categorized into the following five groups (*n* = 5 mice per group): PBS control group, where the mice were intratracheally instilled with PBS and gavaged with vehicle (1% CMC-Na) only; PBS + nintedanib group, where the mice were intratracheally instilled with PBS and gavaged with high-dose nintedanib (60 mg/kg); silica control group, where the mice were intratracheally instilled with silica and gavaged with vehicle; silica + LT group, where the mice were intratracheally instilled with silica and gavaged with low-dose (30 mg/kg) nintedanib; and silica + HT group, where the mice were intratracheally instilled with silica and gavaged with high dose (60 mg/kg) nintedanib. To adhere to the 3R principles (Replacement, Reduction, and Refinement) in animal research, a PBS + low-dose nintedanib group was omitted. The PBS + high-dose group was deemed sufficient to evaluate the potential baseline toxicity of the maximum drug dose in healthy mice.

Mice in the preventive treatment groups were exposed to silica and received nintedanib on the same day for 2 weeks. In the therapeutic experiment, mice were categorized into the following five groups (five per group): PBS, PBS + nintedanib, silica, and silica + LT/HT. These mice received the designated medication daily starting on the 14th day after silica exposure for 2 weeks. Relevant downstream experiments were conducted on the day after drug treatment was completed. The groupings are presented in Table 1.

**Table 1:** Study settings and treatment protocols of the experiments

Group	Preventive					Therapeutic				
PBS or SiO_2_	PBS	PBS	SiO_2_	SiO_2_	SiO_2_	PBS	PBS	SiO_2_	SiO_2_	SiO_2_
Nintedanib dose (mg/kg)	/	60	/	30	60	/	60	/	30	60
Study duration (day)	14	14	14	14	14	14	14	14	14	14
Compound administration (day)	0–14	0–14	0–14	0–14	0–14	14–28	14–28	14–28	14–28	14–28

### Pulmonary function testing

Prior to euthanasia, pulmonary function was assessed in vivo. After anesthetizing the mice with isoflurane, the trachea was exposed anteriorly, and tracheal intubation was performed using a forced lung function test system (Forced Maneuvers by EMMS, UK). The operator conducting these measurements was blinded to the group allocations. We assessed forced murine pulmonary mechanics, including static lung compliance (Cchord, measured in mL/cmH_2_O), total lung capacity (TLC, measured in mL), and airway resistance (measured in cmH_2_O·s/mL).

### Euthanasia, sample collection, and Right Ventricular Hypertrophy Index (RVHI)

Following pulmonary function testing, mice were euthanized under deep anesthesia. The right bronchus was isolated and clamped, and the left lung lobe was lavaged three times with 0.4 mL of normal saline to collect bronchoalveolar lavage fluid (BALF). The BALF samples were placed on ice, centrifuged at 4℃ and 52 g for 5 min within 2 h of collection, and the pellet was used for cell counting. The lungs and heart were then completely excised and separated. The RVHI was calculated using the Fulton index, which is the right ventricle weight divided by the combined weight of the left ventricle and septum [RV/(LV + S)]. The left lung tissues were subsequently snap-frozen in liquid nitrogen or fixed for molecular and histological analyses.

### Cell counting and cytokine detection

#### Cell counting in BALF

The BALF pellet was washed with 400 µL of cold PBS and resuspended. After resuspension, CD16/32 was added to block Fc receptors, and 1 µL of the following antibodies was added: FITC anti-mouse F4/80, APC anti-mouse/human CD11b, PE anti-mouse Ly-6G, and APC/Cyanine7 anti-mouse CD45. Labeling was performed on ice for 30 min. After labeling, 1 mL of PBS was added, followed by centrifugation at 400 g for 5 min. The cells were washed two times with PBS and resuspended in 200 µL of PBS for analysis. The operator analyzing the flow cytometry data was blinded to the sample identities.

#### Quantitative PCR (qPCR)

Total RNA was extracted from lung tissue using TRIzol reagent according to the manufacturer’s instructions. A reverse transcription kit was used to synthesize complementary DNA. qPCR was used to detect gene expression. PCR amplification was performed using the PerfectStart Green qPCR SuperMix (TransGen Biotech, Beijing, China). Data collection and qPCR analyses were performed using a Thermo Fisher ABI-7500 system (Thermo Fisher Scientific, Waltham, MA, USA). The primer sequences are detailed in the Supplementary Materials, and all primers were synthesized by GeneChem Biotech (Shanghai, China).

#### Western blotting

Total protein was extracted from lung tissue using RIPA lysis buffer. Protein concentrations were determined using a BCA protein assay kit. Protein samples were separated on 8%–15% sodium dodecyl sulfate-polyacrylamide gels and transferred to polyvinylidene fluoride membranes. Protein signals were detected using an automated chemiluminescence/fluorescence imaging system. ImageJ software was used to analyze relative protein expression levels. Specifically, the density of each target protein band was first divided by the density of the internal control (β-actin) in the same lane. Importantly, to account for inter-experimental variation, these target/actin ratios were subsequently normalized to the mean ratio of the corresponding PBS control group. The reagents and antibodies used in the experiment were as follows: Anti-Collagen I antibody [3G3] -BSA and Azide free, supplier: abcam, catalogue number: ab88147; Recombinant Anti-Collagen III antibody [EPR17673], supplier: abcam, catalogue number: ab184993; β-Actin (C4) mouse monoclonal IgG1, supplier: Santa Cruz Biotechnology, product number: sc-47,778; Goat anti-Mouse antibody, supplier: IRDye, product number: 925-32210; Goat anti-Rabbit antibody, supplier: IRDye, product number: 925-32211; Precoated protein electrophoresis gel, supplier: GenScript, product number: M00652; Western Blot Blocking Buffer (Protein Free), supplier: TaKaRa, product number: T7132A; Pierced TM RIPA Buffer, supplier: Thermo Scientific, product number: 89,901.

### Lung fibrosis and pathological scoring

The left lung tissue was fixed, embedded in paraffin, and sliced for hematoxylin and eosin (H&E) staining, Masson’s trichrome staining, Sirius red staining, and immunohistochemical detection of collagen I and III. The staining results were obtained after completing the pathological slides. Hematoxylin and eosin (HE) staining was performed to evaluate pulmonary inflammation and quantified using Szapiel’s method [13]. Masson staining was used to reveal the extent of fibrosis in the lung tissues and calculated using King’s method [14]. Specifically, all visible silicon nodules present on the entire section were categorized into five grades based on their distinct structure. Nodules lacking collagen deposition were further classified as grade I, grade II, or grade III, depending on the varying degrees (slight, mild, severe) of inflammatory cell infiltration. Grade IV nodules consisted of collagen fibers accompanied by inflammatory cells, while Grade V nodules were primarily composed of collagen fibers with minimal or absent cell components. To determine the fibrotic score (*FS*) for each Masson image depicting a full pulmonary lobe, nodules ranging from grade I to grade V were assigned scores of 1–5, respectively. The Image-Pro software was utilized to calculate both the area occupied by nodules of various grades (namely, S_I_, S_II,_ S_III_, S_IV_, S_V_) and the total pulmonary lobe area (S_t_). The formula for computing the fibrotic score in each image was as follows. *FS = 1×S*_*I*_*/S*_*t*_ *+2×S*_*II*_*/S*_*t*_*+3×S*_*III*_*/S*_*t*_*+4×S*_*IV*_*/S*_*t*_*+5×S*_*V*_*/S*_*t.*_

For Sirius red staining, collagen deposition was semi-quantified using ImageJ (1.54p, National Institutes of Health, US). Images were split into RGB channels, and the green channel was selected for thresholding. The lower threshold was fixed at approximately half the auto-threshold value (empirically 25 for our acquisition) with the upper limit at 155, applied consistently across all images. The collagen-positive area percentage was calculated as (thresholded area / total field area) × 100. For collagen I immunohistochemistry, color deconvolution (H-DAB preset) was applied to separate the DAB signal. The DAB channel was then thresholded using the Default auto-threshold method (dark background checked), and the COL-I-positive area percentage was calculated similarly.

The HE staining, Masson staining, Sirius red staining and immunohistochemistry of lung tissue were observed and scored by two pathologists in blind mode.

### Statistical methods

All biological and functional assessments were performed with exactly *n* = 5 biological replicates per group. All statistical data were analyzed using the Prism software (version 10.0). Shapiro–Wilk test was used to detect the normality of the data. Comparisons between multiple groups were made using one-way analysis of variance, followed by Tukey’s post-hoc test. All statistical comparisons, including critical baseline comparisons against the PBS control group, are clearly defined in the respective figure legends using standard *P*-value notation (e.g., *P < 0.05 vs. SiO_2_ group). Statistical significance was set at *P* < 0.05.

## Results

### Nintedanib alleviated pulmonary inflammatory in BALF and lung tissue of silicosis mice

#### Neutrophil and macrophage counts in BALF

**Fig. 1:**
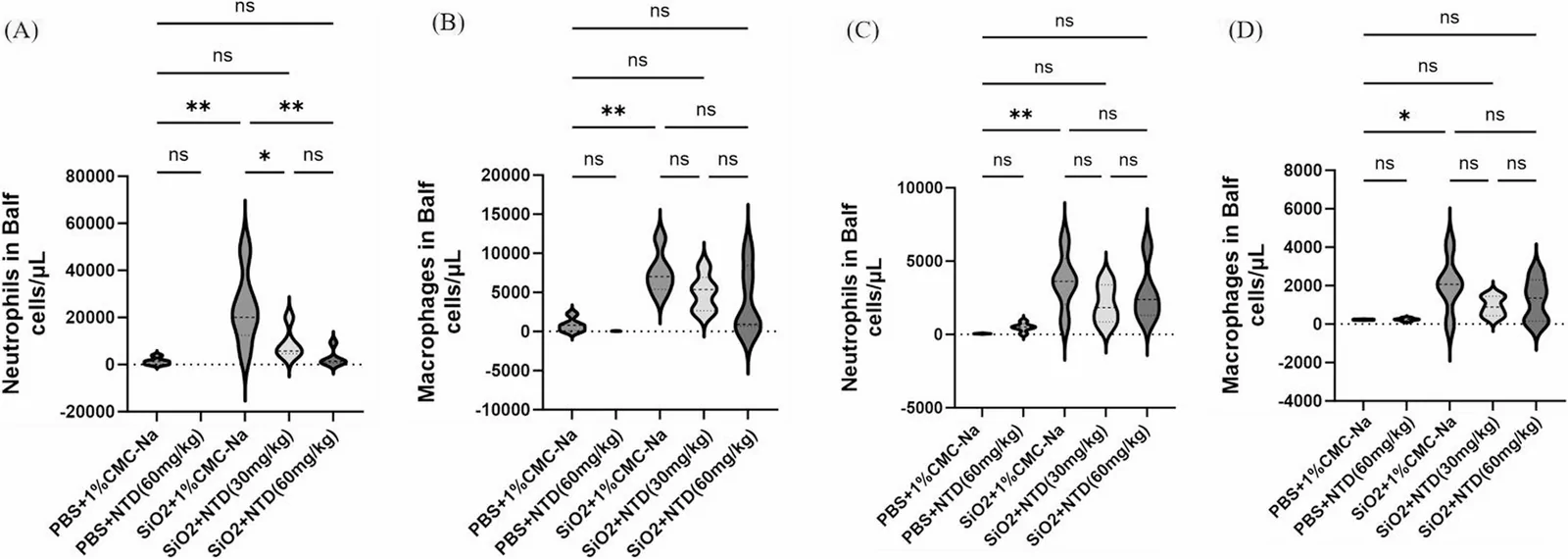
Neutrophil and macrophage counts in bronchoalveolar lavage fluid (BALF). **A** neutrophil count in BALF in the preventive group (5 mice per group); **B**) macrophage count in BALF in the preventive group (5 mice per group); **C** neutrophil count in BALF in the therapeutic group (5 mice per group); **D** macrophage count in BALF in the therapeutic group (5 mice per group). *P < 0.05, **P < 0.01, ***P < 0.001, ns indicates *P* > 0.05

Cell counts in the BALF of mice with silicosis were conducted using flow cytometry. In the preventive intervention model, nintedanib treatment significantly suppressed this inflammatory cell infiltration. Both the low-dose (30 mg/kg) and high-dose (60 mg/kg) nintedanib groups showed a statistically significant reduction in neutrophil counts compared to the SiO_2_ control group (*P* < 0.05 and *P* < 0.01, respectively). However, no statistically significant difference was observed between the two dosage subgroups (*P* > 0.05). Additionally, the preventive group demonstrated a downward trend in macrophage counts, though these reductions did not reach statistical significance when compared to the SiO_2_ control group (*P* > 0.05). In the established fibrosis (therapeutic) model, neutrophil and macrophage counts in the nintedanib-treated groups were generally lower than those in the SiO_2_ control group; however, these reductions did not achieve statistical significance (*P* > 0.05 for all therapeutic treatment subgroups), as shown in Fig. 1.

#### Inflammatory factors in lung tissue

**Fig. 2:**
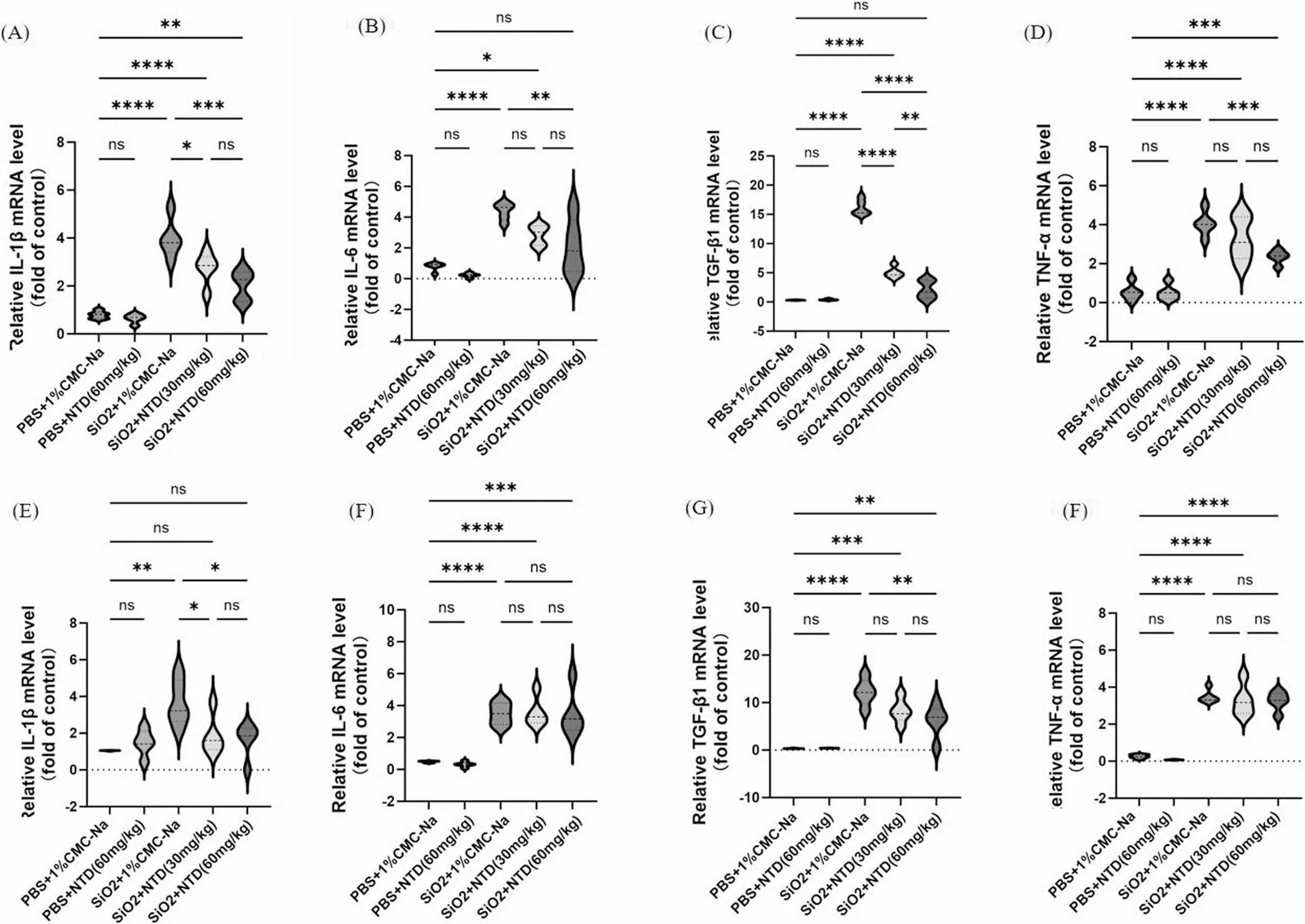
Inflammatory factors in lung tissue. **A**–**D** show the mRNA levels of IL-1β, IL-6, TGF-β1, and TNF-α in lung tissue from the preventive group (5 mice per group). **E**–**H** show the mRNA levels of IL-1β, IL-6, TGF-β1, and TNF-α in lung tissue from the therapeutic group (*n* = 5 per group). *P < 0.05, **P < 0.01, ***P < 0.001, ****P < 0.0001, ns indicates *P* > 0.05. IL-1β, interleukin-1β; IL-6, interleukin-6; TGF-β1, transforming growth factor β1; TNF-α, tumor necrosis factor α

The expression levels of four cytokines in lung tissue were measured using qPCR. Interleukin-1β (IL-1β), interleukin-6 (IL-6), transforming growth factor β1 (TGF-β1), and tumor necrosis factor α (TNF-α) levels decreased in both the preventive and therapeutic groups. In the preventive group, both low- and high-dose treatments of IL-1β and TGF-β1 showed statistical significance. While the low-dose group of IL-6 and TNF-α showed no statistical significance, the high-dose group exhibited a statistically significant difference. In the therapeutic group, IL-1β showed statistical significance in both low- and high-dose treatments, and the high-dose TGF-β1 treatment group also demonstrated statistical significance. However, IL-6 and TNF-α exhibited no significant differences in any of the treatment groups. Details are shown in Fig. 2.

### Nintedanib treatment reduced the RVHI

**Fig. 3:**
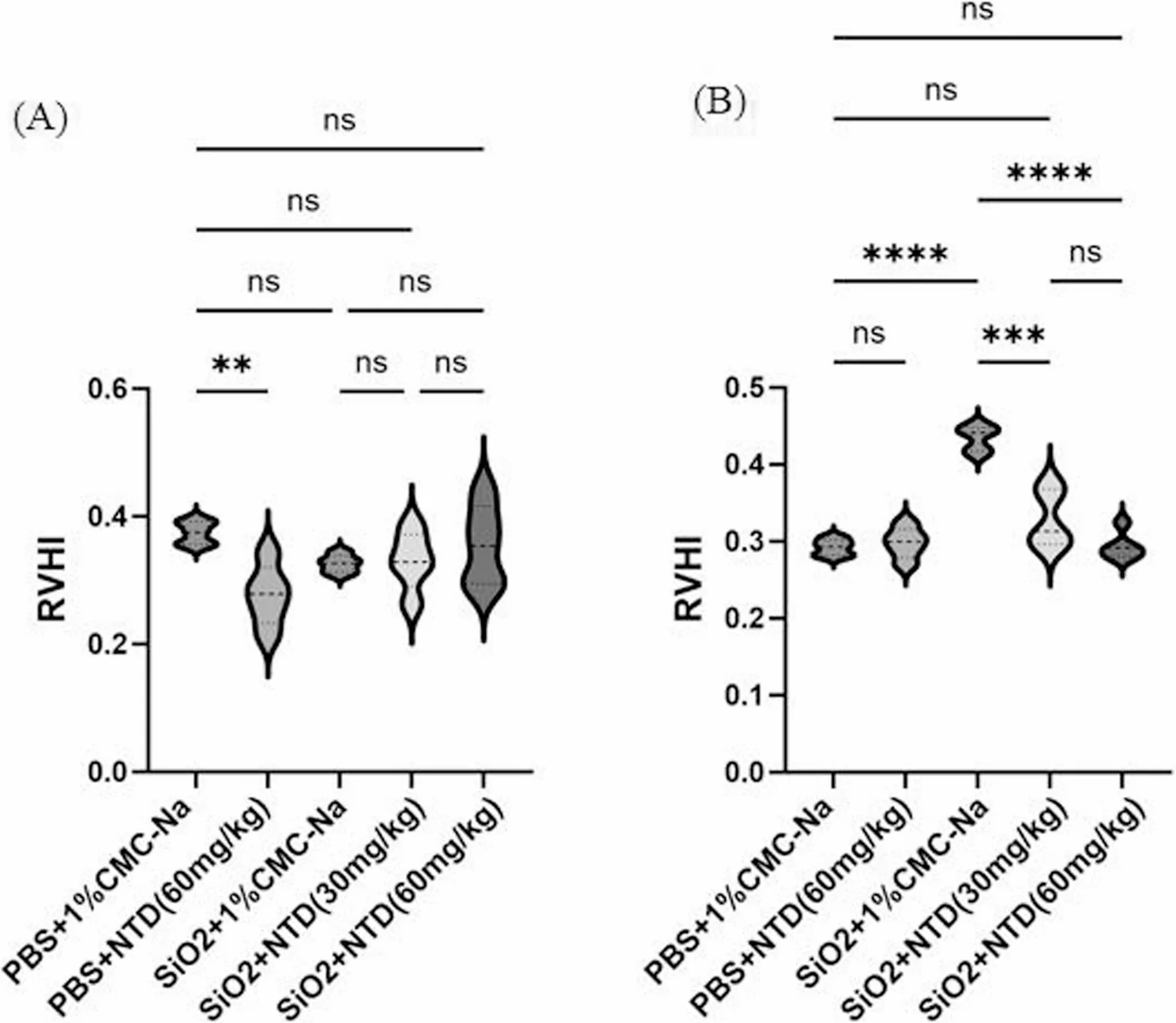
Right ventricular hypertrophy index in mice. **A** shows the RVHI in the preventive group (*n* = 5 per group). **B** shows the RVHI in the therapeutic group (*n* = 5 per group). *P < 0.05, **P < 0.01, ***P < 0.001, ****P < 0.0001, ns indicates *P* > 0.05. RVHI, right ventricular hypertrophy index

In the prevention group, no significant difference was found in the RVHI of the mice. However, in the therapeutic phase, both high- and low-dose nintedanib treatments resulted in a statistically significant decrease in the RVHI compared to the SiO_2_ control (*P* < 0.01 and *P* < 0.001, respectively). Although the reduction appeared more pronounced in the high-dose group, the difference between the two dosages was not statistically significant (*P* > 0.05). (Data are now presented in Fig. 3 to enhance clarity).

### Nintedanib alleviated pulmonary tissue fibrosis

#### Collagen I and collagen III in lung tissue

**Fig. 4:**
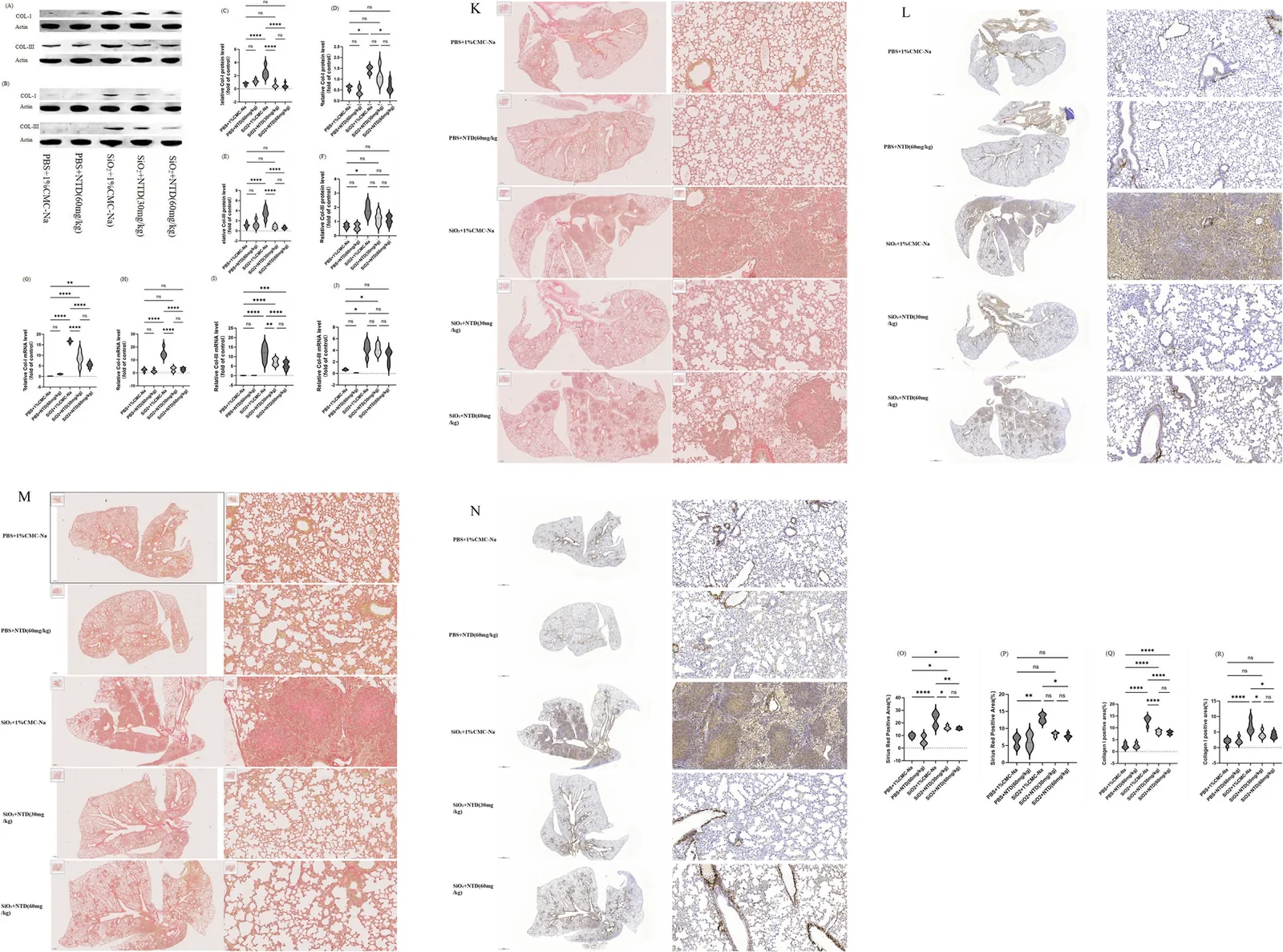
Comparison of collagen I and III in mouse lung tissue. Each experimental group consisted of 5 mice, with quantitative or semi-quantitative data presented as the mean ± SEM of 5 mice per group. For western blot bands and tissue staining images, the most representative sample from each group was selected for display. All tissue staining images were obtained under 2x (left) and 20x (right) magnification microscopy. Panels (**A**) and (**B**) show western blotting bands for collagen I (COL-I) and III (COL-III) in the preventive and therapeutic groups, respectively. Each antibody analyzed represents the western blotting results of a representative animal, with intensity normalized against actin. Panels (**C**) and (**D**) respectively describe the relative expression levels of COL-I protein analyzed by ImageJ software in the prevention group and the treatment group, calculated as gray values/internal control (actin) gray values. Data are expressed as mean ± standard error of the mean (SEM) for five animals per group. Panels (**E**) and (**F**) respectively describe the relative expression levels of COL-III protein analyzed by ImageJ software in the prevention group and the treatment group, calculated as gray values/internal control (actin) gray values. Data are expressed as mean ± standard error of the mean (SEM) for five animals per group. Panels (**G**–**H**) respectively compare collagen I mRNA levels in the preventive group and therapeutic group. Panels (**I**–**J**) respectively compare collagen III mRNA levels in the preventive group and therapeutic group. In panels (**K**), Sirius red staining shows yellow to red collagen I fibers in the preventive group. Panels (**L**) display immunohistochemical images of the same group, with collagen I appearing as distinct brownish-yellow or brown granules. Panels (**M**) and (**N**) show the images of Sirius Red and COL-I for a representative mouse from each therapeutic group. Panels (**O**) and (**P**) show the proportion of the area (%) positive for Sirius red staining in the preventive group and the therapeutic group, respectively. Panels (**Q**) and (**R**) depict the proportion of the area (%) with COL-I in immunohistochemistry staining in the preventive group and the therapeutic group, respectively. *P < 0.05, **P < 0.01, ***P < 0.001, ****P < 0.0001, ns indicates *P* > 0.05

As shown in Fig. 4, we detected the nucleic acid and protein expression of collagen I and Collagen III in lung tissue using QPCR and Western Blot. In the prevention group, both the mRNA and protein expression of collagen I and collagen III were significantly reduced compared to the SiO_2_ control group, with statistically significant differences in both low-dose and high-dose groups. In the treatment group, the mRNA of collagen I was significantly reduced in both low-dose and high-dose groups; however, at the protein level, only the reduction in the high-dose group was statistically significant. The mRNA and protein expression of collagen III showed no statistically significant differences between the low-dose and high-dose groups compared to the SiO_2_ control group.

Similar results were observed with Sirius Red staining and immunohistochemistry. Following treatment with nintedanib, both preventive and therapeutic groups showed a notable reduction in collagen I and III, as illustrated in Fig. 4. In the SiO_2_ group, significant type I collagen deposition was observed, whereas the deposition was markedly reduced in both the low-dose and high-dose treatment groups. In the prevention group, semi-quantitative analysis of Sirius Red staining revealed diminished staining intensity following nintedanib treatment. Consistent with this, immunohistochemistry (IHC) showed decreased deposition of type I collagen after treatment, whereas no statistically significant difference was detected between the high-dose and low-dose groups. In the late-treatment group, the reduction in Sirius Red-positive collagen deposition was restricted to the high-dose subgroup. By contrast, the attenuation of type I collagen deposition reached statistical significance in both the high-dose and low-dose subgroups.

#### Pathological scores for pulmonary fibrosis

**Fig. 5:**
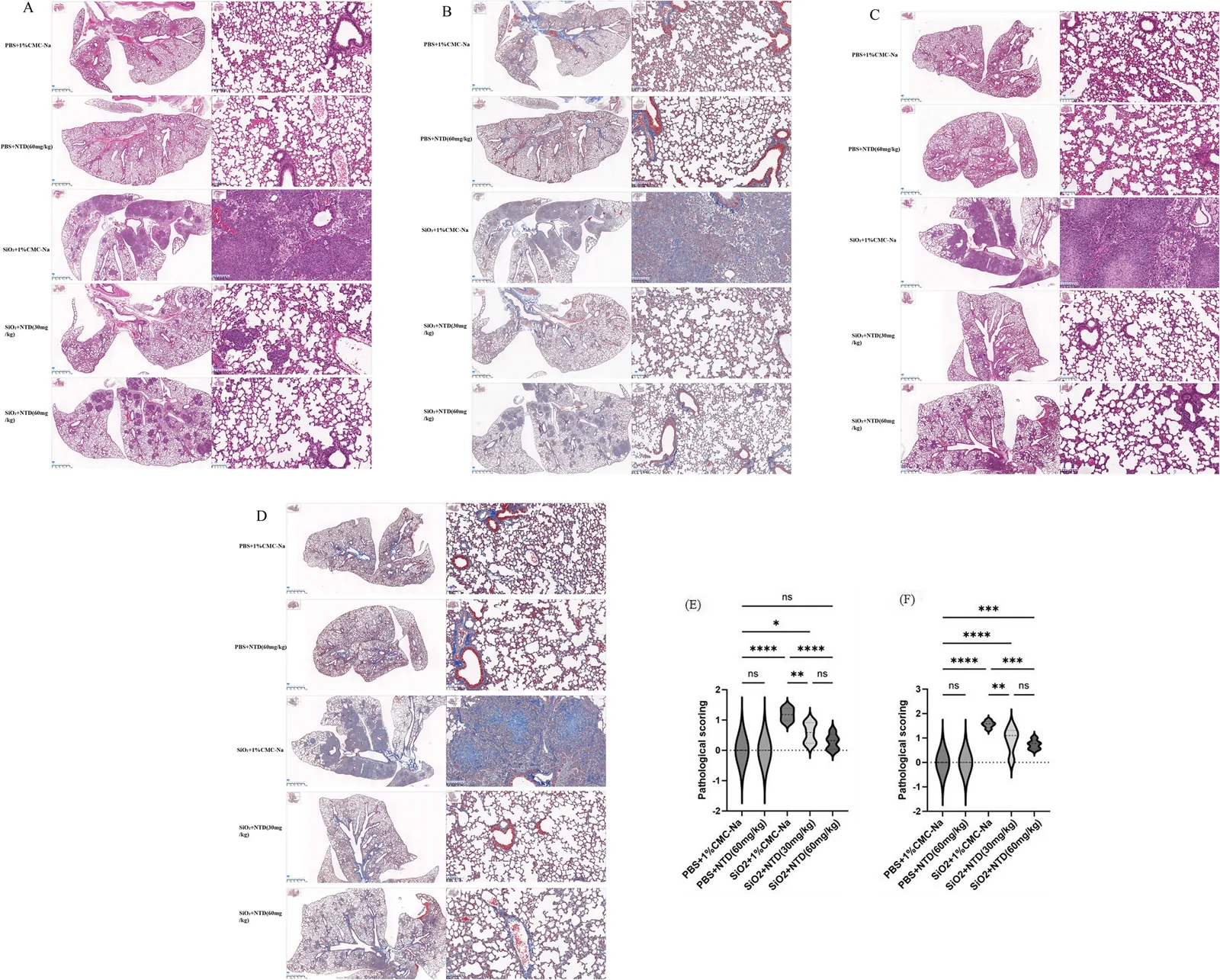
Comparison of hematoxylin and eosin (H&E) staining and Masson’s trichrome staining in mouse lung tissue. We performed staining on 5 mice in each group and selected the most representative one for display. All tissue staining images were obtained under 2x (left) and 20x (right) magnification microscopy. Panels (**A**) and (**B**) show pathological images of H&E and Masson’s trichrome staining in the preventive group. Panels (**C**) and (**D**) depict the pathological images of H&E and Masson’s trichrome staining in the therapeutic group, with each group represented by a single animal. Panels (**E**) and (**F**) classify the fibrotic score (*n* = 5 per group) in the preventive group and the therapeutic group, respectively. *P < 0.05, **P < 0.01, ***P < 0.001, ****P < 0.0001, ns indicates *P* > 0.05. Each experimental group consisted of 5 mice, with scores expressed as the mean ± SEM of pathological scores from 5 mice per group

Histological examination of lung tissue stained with H&E showed a more severe inflammatory response in the silicosis group than in the nintedanib treatment group. Masson’s trichrome staining revealed lower collagen deposition in the nintedanib-treated group, fewer silicotic nodules, and milder pulmonary fibrosis than in the silicosis group, as illustrated in Fig. 5. Furthermore, the high-dose group exhibited less fibrosis than the low-dose group. As shown in Fig. 5, fibrosis scores in the preventive group showed significant differences compared to the SiO_2_ group, regardless of dosage. In the therapeutic phase, the scores in the treatment group decreased, though the reduction was not statistically significant.

#### Pulmonary function testing

**Fig. 6:**
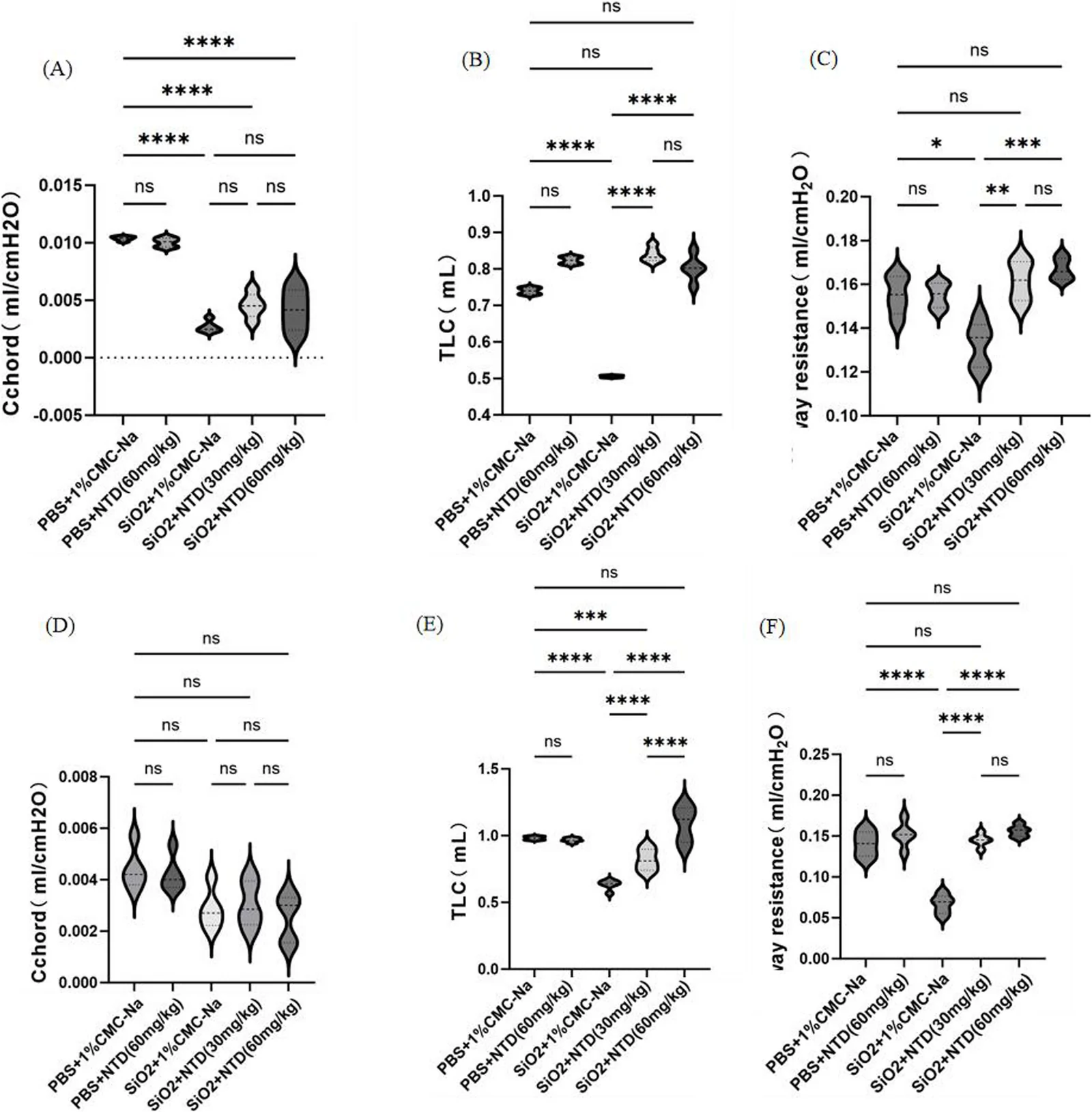
Comparison of lung function in mice. **A**–**C** show Cchord, TLC, and Airway resistance in the preventive group (*n* = 5 per group). **D**–**F** show Cchord, TLC, and Airway resistance in the therapeutic group (*n* = 5 per group). *P < 0.05, **P < 0.01, ***P < 0.001, ****P < 0.0001, ns indicates *P* > 0.05. TLC: total lung capacity

As shown in Fig. 6, exposure to silica significantly deteriorated lung function, evidenced by a dramatic decrease in static lung compliance (Cchord) and total lung capacity (TLC), and a significant increase in airway resistance compared to the PBS baseline (*P* < 0.001). In the preventive group, nintedanib treatment resulted in a statistically significant rescue of TLC (both low- and high-dose) and a significant reduction in airway resistance (high-dose only) compared to the SiO_2_ control. Similarly, in the therapeutic group, both TLC and airway resistance showed statistically significant improvements following nintedanib administration. However, Cchord did not exhibit a significant recovery in either the preventive or therapeutic treatment arms when compared to the respective SiO_2_ control groups (*P* > 0.05).

## Discussion

Nintedanib has been approved for the antifibrotic treatment of idiopathic pulmonary fibrosis and PPF. Preclinical studies have assessed its efficacy and mechanisms using various models, including pulmonary fibrosis induced by bleomycin, silica, chronic allergic inflammation, rheumatoid arthritis, and Fra-2 mutations [15–19]. However, a mouse model of silica-induced pulmonary fibrosis has yet to be evaluated for lung function or ventricular hypertrophy. This study aimed to conduct preclinical assessments using a well-defined mouse model of silicosis. A single intratracheal instillation of SiO_2_ in mice is a reliable method for modeling silicosis, efficiently delivering silica to the lungs. This model exhibits significant inflammatory cell infiltration, severe parenchymal destruction, diffuse pulmonary fibrosis, and fibrous nodules. Histopathological analysis confirmed the increased expression of fibrosis markers, including fibronectin, collagen I, and α-SMA [20].

The results of this study confirmed the pronounced effects of nintedanib on lung inflammation and fibrosis in mice with silicosis.

Initially, nintedanib alleviated the pulmonary inflammatory responses in these mice. Previous studies [21] found that the uptake of silica by alveolar macrophages leads to a cycle of repeated cell phagocytosis, necrosis, silica release, and subsequent cell re-phagocytosis, perpetuating and amplifying the inflammatory process and the release of inflammatory factors [22]. IL-1β is involved in collagen deposition, and IL-1 receptor antagonists can reduce silica and bleomycin-induced pulmonary fibrosis [23]. TNF-α is crucial in silica-induced fibrosis, and anti-TNF treatment can prevent silicosis [24–26]. Quartz-treated human macrophages release insulin-like growth factor and TGF-β, stimulating fibroblast proliferation and collagen synthesis [27]. In this study, after 2 weeks of nintedanib treatment, neutrophils in the BALF were significantly reduced, especially in mice receiving early high-dose treatment. A similar decreasing trend was observed in macrophage counts. These findings are consistent with those reported by Wollin et al. Additionally, mRNA analysis of left lung tissue revealed that nintedanib treatment alleviated lung inflammation in mice. In the preventive group, TNF-α, IL-1β, IL-6, and TGF-β1 levels in lung tissue were lower than those in the silica control group, with the high-dose treatment group showing more significant effects. In the therapeutic group, a notable decrease was found in the expression of IL-1β and TGF-β1, with the high-dose group demonstrating significantly better results than the low-dose group. These findings confirm that nintedanib treatment alleviates pulmonary inflammatory responses in silicotic mice. Furthermore, airway hyperresponsiveness events have been described in previous silicosis mouse models [28, 29], which were found to be associated with increased expression of IL-6 and TNF-α. In this study, we observed reduced expression of IL-6 and TNF-α in the lung tissues of silicosis mice treated with nintedanib. Whether these cytokine reductions translate to functional improvements in airway hyperresponsiveness remains to be directly investigated.

Furthermore, nintedanib alleviated pulmonary fibrosis in mice with silicosis. In this study, we examined collagen I and III in lung tissue, observing notable reductions in the expression and deposition of collagen in silicotic mice following nintedanib treatment compared to positive control mice. Simultaneously, upon analyzing the pathological scores obtained from H&E and Masson staining, the high-dose groups exhibited a reduced score. These results align with those of previous studies by Wollin et al., further confirming the efficacy of nintedanib in pulmonary fibrosis treatment. This effect may be related to the reduction in the pulmonary inflammatory factors described above. Specifically, Nintedanib inhibited neutrophil and macrophage accumulation and suppressed inflammatory factor expression, thereby attenuating the inflammatory response in lung tissue following silica inhalation. The manifestations of fibrosis, such as cell necrosis, collagen deposition, and granuloma formation, were alleviated.

This study also found that nintedanib improved the RVHI. Silicosis significantly increases the risk of pulmonary arterial hypertension (PAH) in patients [30], which can lead to RVH and right heart failure. There has been controversy regarding the effects of nintedanib in treating PAH. Some studies have suggested that it treats PAH through anti-vascular remodeling by directly attenuating pulmonary artery endothelial and smooth muscle cell transformations [31, 32], while others have reported that it does not improve the increased vascular resistance caused by silica dust or right ventricular hypertrophy in mice [33]. Regarding secondary cardiovascular effects, we observed a statistically significant reduction in the right ventricular hypertrophy index (RVHI) following late-stage nintedanib treatment. However, without direct hemodynamic assessments or echocardiographic quantification of vascular remodeling, RVHI must be strictly interpreted as an indirect anatomical marker. We cannot definitively conclude whether this reduction is secondary to relieved pulmonary fibrotic restriction or driven by other unknown systemic mechanisms. To contextualize these findings, it is essential to consider the broader systemic regulation of pulmonary biological functions. Recent groundbreaking studies highlight that pulmonary mesenchymal and vascular homeostasis are deeply governed by metabolic and multikingdom immune cross-talk. For instance, dysregulated lipid metabolism—specifically the accumulation of acylcarnitines—has been shown to directly promote pathological pulmonary mesenchyme proliferation, driving structural remodeling independent of traditional inflammatory pathways [34]. Concurrently, disruptions in the gut microbiome-immune axis, characterized by increased intestinal permeability (“leaky gut”) and endotoxemia, fundamentally trigger systemic Th17 cell dysregulation and severe pulmonary vascular inflammation [35]. Nintedanib’s broad-spectrum inhibition of multiple intracellular tyrosine kinases may indirectly intersect with these complex metabolic and microbial-immune niches, presenting a vital direction for future mechanistic studies.

In terms of pulmonary physiological mechanics, nintedanib administration significantly rescued total lung capacity (TLC) and airway resistance in both experimental phases. Nevertheless, static lung compliance (Cchord) did not exhibit significant physiological recovery compared to the disease model baseline. This selective functional rescue implies that while nintedanib improves certain aspects of lung volume and airway patency, it does not fully restore the restrictive mechanical properties of the fibrotic lung parenchyma, further reflecting the limits of its true anti-fibrotic capacity once tissue architecture is altered.

This study had some limitations. First, the small sample size (*n* = 5 per group) poses a risk of statistical false positives and unstable effect estimates, which requires cautious interpretation. Second, the observation period was relatively short and differed between groups: the preventive group was sampled at 2 weeks post-modeling, while the therapeutic group was sampled at 4 weeks. This may lead to a lack of significant complications, such as pulmonary function impairment and pulmonary hypertension. The inconsistency also precluded direct statistical comparisons between early and late treatments. Third, insufficient dosage of nintedanib: In several instances, the high-dose treatment showed better effects than the low-dose treatment but without statistical significance. Future research should explore higher therapeutic doses to clarify the dose-response relationship. Additionally, while single-dose instillation via the trachea is currently a reliable approach, an uneven distribution of silica between the right and left lungs could introduce bias in the research results [36]. Therefore, future studies should consider different modeling methods to better simulate the state of human inhalation of silica dust more realistically. Fourth, King’s nodule-based scoring system was used for fibrosis. Although sensitive to nodular lesions, it is insensitive to diffuse fibrotic changes (e.g., interstitial thickening, honeycombing), potentially underestimating total fibrosis. Future work using complementary methods like the Ashcroft scale or automated image analysis could address this limitation. Finally, to strictly adhere to the “3R” principles (Replacement, Reduction, and Refinement) in order to minimize animal use, we included only the “PBS + high dose (60 mg/kg)” group to evaluate the potential baseline toxicity of this drug at the maximum dose in healthy mice. However, the absence of a PBS + low-dose nintedanib (30 mg/kg) group represents a limitation: this omission prevents the assessment of the potential effects of low-dose nintedanib on healthy animals and introduces an asymmetry in the experimental design.

## Conclusion

This study demonstrated that nintedanib alleviates lung inflammation and fibrosis in mice with silicosis. Both early treatment, initiated immediately after dust exposure, and late treatment, starting 2 weeks later, exhibited antifibrotic and anti-inflammatory activities.

## Supplementary Information

Supplementary Material 1.

## Data Availability

The datasets used and analysed during the current study are available from the corresponding author on reasonable request.

## Abbreviations

RVHI: right ventricular hypertrophy index
TLC: total lung capacity
SiO_2_: free silica
PPF: progressive pulmonary fibrosis
PBS: phosphate-buffered saline
CMC-Na: sodium carboxymethyl cellulose
BALF: bronchoalveolar lavage fluid
H&E: hematoxylin and eosin
IL-1β: interleukin-1β
IL-6: interleukin-6
TGF-β1: transforming growth factor β1
TNF-α: tumor necrosis factorα
PAH: pulmonary arterial hypertension
